# Prevalence of dementia and BPSD in community-dwelling older adults with advanced care needs: a nationwide survey in Taiwan

**DOI:** 10.1016/j.tjpad.2026.100656

**Published:** 2026-08-14

**Authors:** Kai-Ming Jhang, Yu-Chun Tung, Wen-Fu Wang, Hsiang-Ju Chung, Hui-Yi Liao, Ling-Chun Huang, Chih-Cheng Hsu, Yuan-Han Yang

**Affiliations:** a Neurological Institute, Changhua Christian Hospital, Changhua, Taiwan; b Department of Pharmacy, Taichung Veterans General Hospital, Taichung, Taiwan; c National Center for Geriatrics and Welfare Research, National Health Research Institutes, Yunlin, Taiwan; d Institute of Population Health Sciences, National Health Research Institutes, Maioli, Taiwan; e Department of Health Services Administration, China Medical University, Taichung, Taiwan; f Department of Family Medicine, Min-Sheng General Hospital, Taoyuan, Taiwan; g Department of Neurology, Kaohsiung Medical University Gangshan Hospital, Kaohsiung Medical University, Kaohsiung, Taiwan; h Department of Neurology, Kaohsiung Medical University Hospital, Kaohsiung Medical University, Kaohsiung, Taiwan; i Neuroscience Research Center, Kaohsiung Medical University, Kaohsiung, Taiwan; j School of Post-Baccalaureate Medicine, College of Medicine, Kaohsiung Medical University, Kaohsiung, Taiwan

**Keywords:** Dementia, BPSD, Prevalence, Tube feeding, Enema, Dialysis

## Abstract

**Background:**

Caring for people with both dementia and advanced care needs is complex. This study aimed to elucidate the prevalence of dementia and behavioral and psychological symptoms of dementia (BPSD) in patients receiving advanced care, including tube feeding, respiratory care, subcutaneous injection, urinary catheter, stoma care or enema, wound care, pain care, or dialysis.

**Methods:**

This study was a nationwide, population-based, cross-sectional survey. A total of 11,297 participants were enrolled between September 2020 and June 2022 using a multistage stratified systematic sampling design. Dementia diagnosis was made by subspecialists during in-home visit or by expert consensus. The presence of BPSD and the use of specific advanced care services were collected by trained interviewers.

**Results:**

Overall, 732 (6.5%) participants received at least one item of advanced care and 975 (8.6%) were diagnosed with dementia. Participants receiving advanced care had a higher prevalence of dementia (18.0%vs. 8.0%, *p* < 0.0001). Subjects receiving stoma or enema care (OR=4.92, 95% CI=2.29–10.59, *p* < 0.0001), tube feeding (OR=3.20, 95%CI=1.99–5.15, *p* < 0.0001), dialysis (OR=2.97, 95% CI=1.80–4.92, *p* < 0.0001), urinary catheterization or intermittent catheterization (OR=2.20, 95% CI=1.32–3.67, p = 0.003), and respiratory care (OR=2.01, 95% CI=1.14–3.57, *p* = 0.017) had a significantly higher risk of dementia. For BPSD, day–night confusion (42.0%vs. 27.9%, *p* = 0.002) and care-resistant behaviors (19.8%vs. 12.0%, *p* = 0.019) were significantly more prevalent in participants receiving advanced care.

**Conclusions:**

This study found that patients receiving advanced care had a significantly higher prevalence of dementia and BPSD in Taiwanese population. Healthcare professionals should be vigilant regarding potential comorbid dementia in patients receiving advanced care and provide more patient-centered approaches or non-pharmacological interventions to alleviate BPSD and reduce care partner’s loading.

## Introduction

The global prevalence of dementia is rising, with profound social and economic implications [1]. Taiwan entered a super-aged society in 2025, and the number of patients with dementia has increased dramatically due to the rapid growth of the elderly population. According to the Ministry of Health and Welfare in Taiwan, the prevalence of dementia among community-dwelling adults aged 65 years and older was 7.99% in 2020 [2], which was substantially higher than the 3.7% reported in a regional report in southern Taiwan in 1998 [3].

In addition to cognitive impairment such as memory loss, the management of BPSD poses a greater challenge for both physicians and care partners [4]. A systemic review reported that the prevalence of different BPSD ranged from 4% (elation and mania) to 32% (apathy) in community-dwelling patients with dementia [5]. A small-scale study in Taiwan found that the prevalence of at least one BPSD was high in both community (81.9%) and institutionalized (74.9%) Alzheimer’s disease patients [6]. A nationwide survey of institutional residents in Taiwan found that the prevalence of BPSD was 37.5%, with nighttime behavior, care resistance behavior and depression being the three most common symptoms [7].

With disease progression, people with dementia may develop different advanced care needs, including dysphagia, pain, decreased physical activity, and excretion problems [8]. Dementia is one of the major causes leading to nasogastric tube insertion [9]. Patients receiving life-sustaining treatment such as tube feeding, urinary catheterization, tracheostomy, and hemodialysis also have a high prevalence (38%) of dementia [10]. Chronic constipation is common in older adults with dementia and is also a significant cause of BPSD [11]. People with dementia may also have a higher demand for advanced respiratory care such as sputum suction and oxygen supplementation. Previous studies have demonstrated that patients with chronic obstructive pulmonary disease had a higher risk of dementia, and dementia also increased the risk of aspiration pneumonitis [12,13]. Chronic pain and analgesic use both increase the risk of neurodegenerative disorders [14]. Chronic pain is not only associated with dementia but also with cognitive spectrum disorders such as subjective cognitive decline and mild cognitive impairment [15]. Decreases in physical function were significantly associated with declines in cognitive ability [16]. With dementia progression, pressure injuries are common, and severe dementia was associated with poor healing outcomes [17].

Studies regarding the prevalence of dementia and BPSD in patients with advanced care needs or receiving life-sustaining treatment are limited and fragmented. Most existing studies discussed specific care needs and the prevalence of dementia and were relatively small-scale [18,19]. No studies have compared the prevalence of dementia across different advanced care needs. There are also no studies comparing the prevalence of BPSD in patients receiving advanced care. The present study aims to elucidate the prevalence of dementia and BPSD in patients receiving advanced care (including tube feeding, respiratory care, subcutaneous injection, urinary catheter, stoma care or enema, wound care, pain care, or dialysis) through a large-scale nationwide survey in Taiwan.

## Methods

### Sampling design

This nationwide population-based cross-sectional survey applied a stratified multistage probability proportional to size (PPS) sampling method covering all 22 cities and counties in Taiwan. The sampling population consisted of individuals aged 65 years and older with a registered residence in Taiwan as of April 30, 2020. Participants were interviewed between September 2020 and June 2022. The sampling method considered the demographic structure, medical resources, land use, livelihood economy, and industrial development of each township or district in each city or county, derived from a series of surveys conducted by the Taiwanese government. Based on the PPS principle, there were three levels of sampling units: the primary sampling unit was the township or district, secondary was the village or “Li” (both are official administrative divisions in Taiwan), and the tertiary unit was the individual. Any participant residing in a long-term care institution was replaced with an alternative sample.

### Study procedures

This study ascertained dementia diagnoses in two phases. In phase 1, trained interviewers conducted in-person interviews at the participants' homes. The structured questionnaire in this phase included basic demographic data, comorbidities, advanced care needs, behavioral and psychological symptoms of dementia (BPSD), the eight-item informant interview to Differentiate Aging and Dementia (AD8), the Mini-Mental State Examination (MMSE), and the 5-item Geriatric Depression Scale (GDS-5). All interviewers were trained by experienced physicians with expertise in dementia and by a senior case manager in long-term care. Individuals suspected of having dementia (MMSE <26, AD8≥2, or self-reported history of dementia) during phase 1 survey were further evaluated in phase 2.

In phase 2, psychiatrists or neurologists conducted home visits to determine the diagnosis and severity of dementia using the National Institute of Neurological and Communicative Disorders and Stroke/Alzheimer's Disease and Related Disorders Association (NINCDS-ADRDA) diagnostic criteria and the Clinical Dementia Rating (CDR) scale [20,21]. All physicians were required to complete a series of standardized training courses on CDR scoring standards. Computer-based logic checks were performed by monitors in the research team to ensure the quality and reliability of the data collected from phase 1 and phase 2 surveys. Ultimately, this survey successfully collected data from a total of 11,297 participants through the collaborative efforts of interviewers and the research team.

### Definition of individuals with dementia

**Figure 1:**
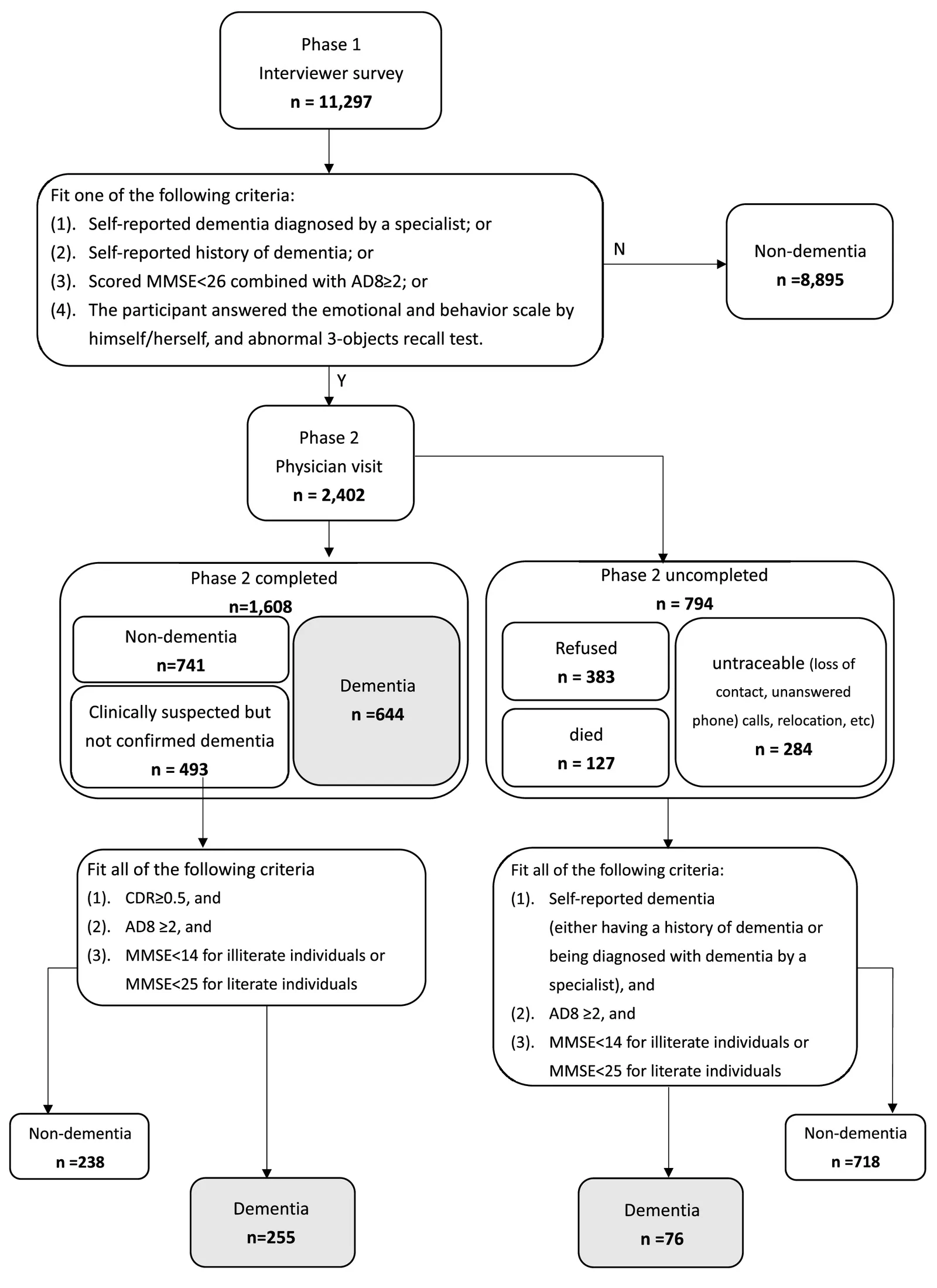
Flowchart of diagnosis of dementia.

Fig. 1 shows the diagnostic flowchart. A total of 2402 participants met the criteria for a phase 2 visit. For individuals evaluated by a physician in phase 2 (n = 1608), dementia was defined as follows: (1) physician-confirmed diagnosis of dementia; or (2) physician-reported clinically suspected but unconfirmed dementia, with a CDR score≥0.5, and AD8≥2, and MMSE<14 for illiterate individuals, or MMSE<25 for literate individuals. A total of 794 participants (33.1%) did not complete the phase 2 visit, including 383 who refused due to the COVID-19 pandemic, 127 who died, and 284 who were untraceable (loss of contact, unanswered phone calls, relocation, etc.). For individuals unable to complete the phase 2 evaluation, dementia was defined if all three of the following criteria were met: (1) self-reported history of dementia (either a previous diagnosis or diagnosed by a specialist); (2) AD8≥2; and (3) MMSE<14 for illiterate individuals or MMSE<25 for literate individuals. Difficult cases were reviewed and discussed by an expert panel consisting of experienced neurologists and psychiatrists, who made a final diagnosis based on consensus.

### Definition of advanced care and behavior or psychological symptoms

Eight kinds of advanced care were recorded, including tube feeding (nasogastric tube or gastrostomy feeding), respiratory care (tracheostomy, ventilation, oxygen supplementation, or sputum suction), subcutaneous injection (eg., insulin or erythropoiesis-stimulating agent), urinary catheter or single catheterization, stoma care or enema, wound or draining tube care (including superficial wounds and pressure sores), pain care (including the use of analgesics, physical therapy, or any non-invasive pain relief strategies such as mind-body techniques), and hemodialysis or peritoneal dialysis.

Care partner-reported behavior or psychological symptoms were included in the analysis, including wandering, day–night confusion or nighttime behavior, agitation or aggression (including verbal aggression such as offensive language; physical aggression including attacks on people or objects; other disruptive or interfering behavior), care-resistant behavior (defined as refusal of care or non-cooperation with the care partner), delusion, hallucination, fear or anxiety, depression or suicidal attempt, aberrant motor behavior, and socially inappropriate behavior.

### Statistical analysis

For continuous variables, results are presented as mean and standard deviation. For categorical variables, results are presented as the number of individuals and percentage (%). Differences between variables were assessed using the chi-squared test for categorical variables and the Mann-Whitney U test for continuous variables. Multivariable logistic regression models were applied to determine the risk of dementia in participants receiving advanced care. All tests were two-sided and p-values< 0.05 were considered statistically significant. All analyses were carried out using SAS 9.4 (SAS Institute Inc., Cary, NC)

### Statement of ethics

The study protocol was approved by the Ethics Review Board of the National Health Research Institutes (IRB No.EC1090409). Written informed consent was obtained from all participants. Adherence to the principles delineated in the Declaration of Helsinki was assured in all research involving human participants.

## Result

A total of 11,297 subjects completed the phase 1 interviewer survey. Among them, 732 (6.5%) participants received at least one type of advanced care. Table 1 presents the demographic characteristics. Participants with advanced care were older (74.4 ± 7.3 vs. 77.1 ± 8.1 years, *p* < 0.0001), had lower educational level, and had more comorbidities, including hypertension (HTN), diabetes mellitus (DM), and cardiovascular or cerebrovascular diseases, than those without advanced care. Subjects with advanced care had worse cognitive performance (MMSE: 24.3 ± 7.0 vs. 18.4 ± 11.1, *p* < 0.0001; AD8: 1.2 ± 1.8 vs. 1.8 ± 2.4, *p* < 0.0001) and more neuropsychiatric symptoms, including day–night confusion (5.2%vs. 19.1%, *p* < 0.0001), agitation or aggression (3.5%vs. 8.4%, *p* < 0.0001), care-resistant behaviors (2.0%vs. 5.9%, *p* < 0.0001), delusions (3.2%vs. 8.6%, *p* < 0.0001), hallucinations (2.9%vs. 7.9%, *p* < 0.0001), fear or anxiety (8.7%vs 15.9%, *p* < 0.0001), depression or suicidal attempt (8.7%vs. 19.5%, *p* < 0.0001; GDS-5: 0.5 ± 1.1 vs 0.9 ± 1.5, *p* < 0.0001), aberrant motor behavior (4.5%vs. 11.1%, *p* < 0.0001), and socially inappropriate behavior (0.5%vs. 1.5%, *p* = 0.001).

**Table 1:** Basic Characteristics of participants with or without advanced care.

	Without advanced care (n = 10,565)	With advanced care (n = 732)	p value
	n (%)	n (%)	
Sex			0.704^f^
Male	4876 (46.2)	332 (45.4)	
Female	5689 (53.8)	400 (54.6)	
Age, Mean(SD)	74.4 (7.3)	77.1 (8.1)	<0.0001 *g*^g^
Education^a^			<0.0001^f^
Illiteracy	1646 (15.6)	152 (20.9)	
1–6 years	4876 (46.2)	363 (50.0)	
7–12 years	2808 (26.6)	162 (22.3)	
≥13 years	1226 (11.6)	49 (6.7)	
Social Welfare^b^			0.139 f
General	10,185 (96.8)	694 (95.7)	
Low income	336 (3.2)	31 (4.3)	
Comorbidities			
HTN	5511 (52.2)	441 (60.2)	<0.0001^f^
DM	2543 (24.1)	288 (39.3)	<0.0001^f^
Cardiovascular disease	1396 (13.2)	144 (19.7)	<0.0001^f^
Cerebrovascular disease	409 (3.9)	83 (11.3)	<0.0001^f^
MMSE scores, Mean(SD)	24.3 (7.0)	18.4 (11.1)	<0.0001 *g*^g^
AD8 scores, Mean(SD)	1.2 (1.8)	1.8 (2.4)	<0.0001 *g*^g^
GDS-5 scores, Mean(SD)^c^	0.5 (1.1)	0.9 (1.5)	<0.0001 *g*^g^
Behavior or psychological symptoms^d^			
Wandering	119 (1.2)	13 (1.8)	0.154^f^
Day night confusion	538 (5.2)	136 (19.1)	<0.0001^f^
Agitation or aggression	357 (3.5)	60 (8.4)	<0.0001^f^
Verbal aggression	298 (2.9)	51 (7.2)	<0.0001^f^
Physical aggression	94 (0.9)	16 (2.2)	0.001^f^
Other disruptive behavior	111 (1.1)	20 (2.8)	<0.0001^f^
Care-resistant behaviors	208 (2.0)	42 (5.9)	<0.0001^f^
Delusion^e^	327 (3.2)	61 (8.6)	<0.0001^f^
Hallucination^e^	304 (2.9)	56 (7.9)	<0.0001^f^
Fear or anxiety	895 (8.7)	113 (15.9)	<0.0001^f^
Depression or suicidal attempt	902 (8.7)	139 (19.5)	<0.0001^f^
Aberrant motor behavior	462 (4.5)	79 (11.1)	<0.0001^f^
Socially inappropriate behavior	49 (0.5)	11 (1.5)	0.001^h^

SD=standard deviation; HTN=hypertension; DM=diabetes mellitus; MMSE=mini-mental status examination; AD8= The Ascertain Dementia 8-item questionnaire; GDS=geriatric depression scale.

a Exclude 15 subjects reported unknown.

b Exclude 51 subjects reported unknown.

c Exclude 621 subjects refused or unable to answer the questionnaire.

d Exclude 248 participants without care partner.

e Exclude 249 participants without care partner or refused to answer.

f Chi-square test.

g Mann-Whitney U test.

h Fisher's Exact test.

A total of 975 individuals with dementia were identified among the 11,297 participants, including 644 subjects diagnosed with dementia by physicians in the phase 2 interview, 255 after the consensus made by the expert panel, and 76 subjects who refused phase 2 interview or were untraceable during the COVID-19 pandemic and were diagnosed based on phase 1 questionnaires only (Fig. 1). Table 2 shows the prevalence of dementia in participants with or without advanced care. Participants receiving advanced care had higher prevalence of dementia (8.0%vs.18.0%, *p* < 0.0001). Participants receiving each of eight specific advanced care items had a significantly higher prevalence of dementia compared with those not receiving advanced care. The prevalence of dementia also increased with an increasing number of advanced care items (8.0%, 17.8%, 31.3%, and 29.6% for participants receiving 0, 1, 2, and ≥3 items of advanced care, respectively).

**Table 2:** Prevalence of dementia in participants with or without advanced care.

	Not demented	Dementia	p value
	(n = 10,322)	(n = 975)	
	n (%)	n (%)	
Without advanced care	9722 (92.0)	843 (8.0)	<0.0001
With any advanced care	600 (82.0)	132 (18.0)	
Item of advanced care			
Tube feeding	55 (59.8)	37 (40.2)	<0.0001∫
Respiratory care*	59 (75.6)	19 (24.4)	<0.0001∫
Subcutaneous injection	92 (85.2)	16 (14.8)	<0.0001∫
Urinary catheter or single catheterization	58 (69.9)	25 (30.1)	<0.0001∫
Stoma care or enema	19 (57.6)	14 (42.4)	<0.0001∫
Wound or draining tube care	67 (83.8)	13 (16.3)	<0.0001∫
Pain care	155 (87.6)	22 (12.4)	<0.0001∫
Hemodialysis or Peritoneal dialysis	82 (78.1)	23 (21.9)	<0.0001∫
Numbers of advanced care item#			<0.0001
0	9840 (92.0)	853 (8.0)	
1	406 (82.2)	88 (17.8)	
2	57 (68.7)	26 (31.3)	
≥3	19 (70.4)	8 (29.6)	

# Using how many kinds of advanced care (total eight items: tube feeding, respiratory care, subcutaneous injection, urinary catheter or single catheterization, stoma care or enema, wound or draining tube care, pain care, or dialysis).

∫ Compared with not receiving specific items of advanced care.

⁎ Tracheostomy or ventilation or oxygen supplement or sputum suction.

Table 3 presents the odds ratio (OR) for dementia in participants receiving advanced care. Logistic regression models adjusted for age, sex, education level, welfare status, comorbidities were applied. Participants receiving any advanced care were associated with a higher risk of dementia (OR=1.74, 95% CI=1.40–2.16, *p* < 0.0001). Subjects receiving tube feeding (OR=3.20, 95%CI=1.99–5.15, *p* < 0.0001), respiratory care (OR=2.01, 95% CI=1.14–3.57, *p* = 0.017), urinary catheterization or single catheterization (OR=2.20, 95% CI=1.32–3.67, *p* = 0.003), stoma care or enema (OR=4.92, 95% CI=2.29–10.59, *p* < 0.0001), and hemodialysis or peritoneal dialysis (OR=2.97, 95% CI=1.80–4.92, *p* < 0.0001) had significantly higher risks of dementia. For participants receiving subcutaneous injection, wound or draining tube care, and pain care, there was a non-significantly trend toward an increased risk of dementia. The number of advanced care items also predicted a higher risk of dementia (one item: OR=1.86, 95% CI 1.44–2.41, *p* < 0.0001; two items: OR=2.41, 95% CI=1.44–4.04, *p* = 0.001; ≥3 items, OR=2.39, 95% CI=0.96–5.94, *p* = 0.062).

**Table 3:** Logistic regression model to predict risk of dementia in participants receiving advanced care*.

	OR (95%CI)	p value
With any advanced care (Ref. without)	1.74 (1.40–2.16)	<0.0001
Item of advanced care (Ref. without any advanced care)		
Tube feeding	3.20 (1.99–5.15)	<0.0001
Respiratory care	2.01 (1.14–3.57)	0.017
Subcutaneous injection	1.67 (0.94–2.97)	0.081
Urinary catheter or single catheterization	2.20 (1.32–3.67)	0.003
Stoma care or enema	4.92 (2.29–10.59)	<0.0001
Wound or draining tube care	1.30 (0.69–2.43)	0.421
Pain care	1.28 (0.79–2.06)	0.310
Hemodialysis or Peritoneal dialysis	2.97 (1.80–4.92)	<0.0001
Numbers of advanced care item (Ref: 0)		
1	1.86 (1.44–2.41)	<0.0001
2	2.41 (1.44–4.04)	0.001
≥3	2.39 (0.96–5.94)	0.062

*Each model adjusted age, sex, education, welfare, comorbidities (HTN, DM, Cardiovascular disease, Cerebrovascular disease).

Table 4 presents the prevalence of BPSD in participants with and without advanced care. Among the 975 subjects diagnosed with dementia, eight participants were excluded because BPSD data were self-reported by patients themselves. Day–night confusion (27.9%vs. 42.0%, *p* = 0.002) and care-resistant behaviors (12.0% vs. 19.8%, *p* = 0.019) were significantly more prevalent in participants receiving advanced care. Table 5 presents the prevalence of BPSD among participants receiving specific types of advanced care. The number of subjects with dementia receiving specific advanced care was relatively small, resulting in variable BPSD prevalence estimates. The prevalence of day–night confusion was higher in participants receiving a urinary catheter or undergoing single catheterization (56%vs 27.9%, *p* = 0.005). Care-resistant behavior was more prevalent in individuals receiving tube feeding (27%vs 12%, *p* = 0.018). Patients receiving dialysis tended to have a higher prevalence of hallucination (39.1%vs 18.4%, *p* = 0.026) and aberrant motor behavior (47.8%vs 25%, *p* = 0.026). Aberrant motor behavior was less frequent in patients receiving tube feeding (8.1%vs 25%, *p* = 0.032).

**Table 4:** Prevalence of BPSD in participants with or without advanced care.

	Without advanced care (n = 836)	With advanced care (n = 131)	p value
	n (%)	n (%)	
Wandering	72 (8.6)	6 (4.6)	0.161
Day night confusion	233 (27.9)	55 (42.0)	0.002
Agitation or aggression	154 (18.4)	24 (18.3)	1.000
Verbal aggression	123 (14.7)	18 (13.7)	0.873
Physical aggression	52 (6.2)	9 (6.9)	0.927
Other disruptive behavior	70 (8.4)	12 (9.2)	0.895
Care-resistant behaviors	100 (12.0)	26 (19.8)	0.019
Delusion	171 (20.5)	28 (21.4)	0.905
Hallucination	154 (18.4)	28 (21.4)	0.498
Fear or anxiety	239 (28.6)	31 (23.7)	0.288
Depression or suicidal attempt	274 (32.8)	46 (35.1)	0.668
Aberrant motor behavior	209 (25.0)	35 (26.7)	0.755
Socially inappropriate behavior	37 (4.4)	4 (3.1)	0.623

**Table 5:** Prevalence of BPSD in participant receiving specific advanced care.

	Without advanced care (n = 836)		Tube (n = 37)			Trachea (n = 19)			Inject (n = 16)			Foley (n = 25)		
	n (%)	95% CI	n (%)	95% CI	p value&	n (%)	95% CI	p value&	n (%)	95% CI	p value&	n (%)	95% CI	p value&
Wandering	72 (8.6)	6.8–10.7	0 (0.0)	―	―	1 (5.3)	0.1–26.0	1.000∫	0 (0.0)	―	―	0 (0.0)	―	―
Day night confusion	233 (27.9)	24.9–31.0	15 (40.5)	24.8–57.9	0.137∫	9 (47.4)	24.5–71.1	0.108∫	5 (31.3)	11.0–58.7	0.781∫^,^∫	14 (56.0)	34.9–75.6	0.005∫
Agitation or aggression	154 (18.4)	15.9–21.2	6 (16.2)	6.2–32.0	0.903∫	3 (15.8)	3.4–39.6	1.000∫	2 (12.5)	1.6–38.4	0.750∫	6 (24.0)	9.4–45.1	0.441^∫^
Verbal aggression	123 (14.7)	12.4–17.3	3 (8.1)	1.7–21.9	0.379∫	1 (5.3)	0.1–26.0	0.339∫	2 (12.5)	1.6–38.4	1.000∫	4 (16.0)	4.5–36.1	0.777∫
Physical aggression	52 (6.2)	4.7–8.1	2 (5.4)	0.7–18.2	1.000∫	3 (15.8)	3.4–39.6	0.117∫	1 (6.3)	0.2–30.2	1.000∫	3 (12.0)	2.6–31.2	0.210∫
Other disruptive behavior	70 (8.4)	6.6–10.5	2 (5.4)	0.7–18.2	0.761∫	1 (5.3)	0.1–26.0	1.000∫	1 (6.3)	0.2–30.2	1.000∫	2 (8.0)	1.0–26.0	1.000∫
Care-resistant behaviors	100 (12.0)	9.8–14.4	10 (27.0)	13.8–44.1	0.018∫	3 (15.8)	3.4–39.6	0.491∫	2 (12.5)	1.6–38.4	1.000∫	4 (16.0)	4.5–36.1	0.530∫
Delusion	171 (20.5)	17.8–23.4	5 (13.5)	4.5–28.8	0.410∫	3 (15.8)	3.4–39.6	0.778∫	1 (6.3)	0.2–30.2	0.217∫	7 (28.0)	12.1–49.4	0.507∫
Hallucination	154 (18.4)	15.9–21.2	8 (21.6)	9.8–38.2	0.787∫	3 (15.8)	3.4–39.6	1.000∫	0 (0.0)	―	―	6 (24.0)	9.4–45.1	0.441∫
Fear or anxiety	239 (28.6)	25.6–31.8	9 (24.3)	11.8–41.2	0.707^∫^	5 (26.3)	9.2–51.2	1.000∫	2 (12.5)	1.6–38.4	0.260∫	7 (28.0)	12.1–49.4	1.000∫
Depression or suicidal attempt	274 (32.8)	29.6–36.1	11 (29.7)	15.9–47.0	0.836∫	4 (21.1)	6.1–45.6	0.406∫	5 (31.3)	11.0–58.7	1.000∫	8 (32.0)	15.0–53.5	1.000∫
Aberrant motor behavior	209 (25.0)	22.1–28.1	3 (8.1)	1.7–21.9	0.032∫	1 (5.3)	0.1–26.0	0.057∫	4 (25.0)	7.3–52.4	1.000∫	4 (16.0)	4.5–36.1	0.428∫
Socially inappropriate behavior	37 (4.4)	3.1–6.1	2 (5.4)	0.7–18.2	0.679∫	0 (0.0)	―	―	1 (6.3)	0.2–30.2	0.521∫	0 (0.0)	―	―

	Without advanced care (n = 836)		Stool (n = 14)			Wound (n = 13)			Pain (n = 22)			Dialysis (n = 23)		
	n (%)	95% CI	n (%)	95% CI	p value&	n (%)	95% CI	p value&	n (%)	95% CI	p value&	n (%)	95% CI	p value&
Wandering	72 (8.6)	6.8–10.7	1 (7.1)	0.2–33.9	1.000∫	2 (15.4)	1.9–45.5	0.315∫	1 (4.5)	0.1–22.8	1.000∫	1 (4.3)	0.1–22.0	0.713∫
Day night confusion	233 (27.9)	24.9–31.0	6 (42.9)	17.7–71.1	0.235∫	3 (23.1)	5.0–53.8	1.000∫	6 (27.3)	10.7–50.2	1.000∫	9 (39.1)	19.7–61.5	0.343∫
Agitation or aggression	154 (18.4)	15.9–21.2	4 (28.6)	8.4–58.1	0.308∫	4 (30.8)	9.1–61.4	0.277∫	4 (18.2)	5.2–40.3	1.000∫	4 (17.4)	5.0–38.8	1.000∫
Verbal aggression	123 (14.7)	12.4–17.3	1 (7.1)	0.2–33.9	0.706∫	4 (30.8)	9.1–61.4	0.116∫	4 (18.2)	5.2–40.3	0.553∫	2 (8.7)	1.1–28.0	0.559∫^,^∫
Physical aggression	52 (6.2)	4.7–8.1	3 (21.4)	4.7–50.8	0.056^∫^	2 (15.4)	1.9–45.5	0.198∫	1 (4.5)	0.1–22.8	1.000∫	0 (0.0)	―	―
Other disruptive behavior	70 (8.4)	6.6–10.5	2 (14.3)	1.8–42.8	0.335∫	3 (23.1)	5.0–53.8	0.093∫^,^∫	2 (9.1)	1.1–29.2	0.706∫	2 (8.7)	1.1–28.0	1.000∫
Care-resistant behaviors	100 (12.0)	9.8–14.4	3 (21.4)	4.7–50.8	0.235∫	4 (30.8)	9.1–61.4	0.063∫	4 (18.2)	5.2–40.3	0.328∫	4 (17.4)	5.0–38.8	0.510∫
Delusion	171 (20.5)	17.8–23.4	2 (14.3)	1.8–42.8	0.747∫	3 (23.1)	5.0–53.8	0.736∫	6 (27.3)	10.7–50.2	0.427∫	6 (26.1)	10.2–48.4	0.600∫
Hallucination	154 (18.4)	15.9–21.2	3 (21.4)	4.7–50.8	0.731∫	4 (30.8)	9.1–61.4	0.278∫	4 (18.2)	5.2–40.3	1.000∫	9 (39.1)	19.7–61.5	0.026∫
Fear or anxiety	239 (28.6)	25.6–31.8	2 (14.3)	1.8–42.8	0.371∫	2 (15.4)	1.9–45.5	0.370∫	6 (27.3)	10.7–50.2	1.000∫	3 (13.0)	2.8–33.6	0.162∫
Depression or suicidal attempt	274 (32.8)	29.6–36.1	2 (14.3)	1.8–42.8	0.247∫	2 (15.4)	1.9–45.5	0.241∫	11 (50.0)	28.2–71.8	0.143∫	11 (47.8)	26.8–69.4	0.198∫
Aberrant motor behavior	209 (25.0)	22.1–28.1	3 (21.4)	4.7–50.8	1.000∫	3 (23.1)	5.0–53.8	1.000∫	7 (31.8)	13.9–54.9	0.632∫	11 (47.8)	26.8–69.4	0.026∫
Socially inappropriate behavior	37 (4.4)	3.1–6.1	0 (0.0)	―	―	1 (7.7)	0.2–36.0	0.451∫	0 (0.0)	―	―	0 (0.0)	―	―

& Chi-square test or Fisher's Exact test.

∫ Compared with without advanced care.

## Discussions

To the best of our knowledge, this is the first large-scale study to demonstrate the prevalence of dementia and BPSD in patients receiving advanced care among community-dwelling older adults. Patients with advanced care needs had a higher prevalence of dementia. Tube feeding, respiratory care, urinary catheterization, stoma care or enema, and dialysis were associated with a significantly higher risk of dementia. Patients with advanced care needs also exhibited more neuropsychiatric symptoms, including day–night confusion, agitation or aggression, care-resistant behaviors, delusions, hallucinations, fear or anxiety, depression or suicidal attempt, aberrant motor behavior, and socially inappropriate behavior. Among participants with dementia, day–night confusion and care-resistant behaviors were significantly more prevalent in the advanced care group.

Huang et al. analyzing home healthcare services users (most of whom receiving life-sustaining treatments such as tube feeding, urinary catheterization, tracheostomy and hemodialysis) in Taiwan reported that the prevalence of dementia was 38% [10]. The present study also found a high prevalence of dementia among patients receiving advanced care, with 21.9%, 24.4%, 30.1%, and 40.2% in patients undergoing dialysis, respiratory care, urinary catheterization, and tube feeding, respectively. Another report using U.S. Medicare claims data found that 1.3–2.9% of people with dementia had a feeding tube placed in the last 30 days of life, with higher percentages among Black and Hispanic than White decedents [22]. The frequency of tube feeding among people with dementia in the present survey was 3.8% (37/975), which was higher than that reported in the United States. Dementia and dysphagia are bidirectional. Advanced dementia causes dysphagia, while tube insertion also precipitates delirium and may accelerate cognitive decline [55]. Clinically assisted nutrition for people with advanced dementia requires both scientific and ethical justification, and socio-familial factors are important dimensions influencing decision-making [23]. Susan et al. reported among people with advanced dementia receiving home care in the United States, 11.9% had a feeding tube, 15.8% had Foley catheter, 12.5% used oxygen therapy, 19.7% had pressure ulcer, and 53.4% reported almost daily pain [24]. Taiwanese society is strongly influenced by the traditional value of filial piety. Intergenerational caregiving behaviors are complex and influenced by the relationship between care partners and care recipients as well as by caregiving resources [49]. The Long-Term Care Plan in Taiwan is a tax-funded, universal system delivering person-centered, community-based, and continuous care at home or in the community in order to support aging in place [50]. People with dementia aged 50 and over are covered by long-term care services. The accessibility of home healthcare resources is also high in Taiwan, especially for those with advanced care needs [51]. These specific care environments all influence advanced care choices among people with dementia. Although it is difficult to directly compare percentages across studies in different countries, the correlation between dementia and advanced care needs appears to be substantial.

Stoma or enema care had the highest prevalence of dementia in the present study (42.4%, OR=4.92 compared with those not receiving any advanced care), with 1.4% (14/975) of people with dementia requiring stoma or enema care at home. To date, no studies have reported the prevalence of dementia among community-dwelling individual requiring enema or stoma. One large retrospective cohort study revealed that constipation was associated with a 50% and 56% increased incidence of all-cause dementia and Parkinson’s disease, respectively [25]. Another prospective study found that regular use of multiple types of laxative or osmotic laxative increased the risk of all-cause dementia [26]. A population-based cross-sectional survey in China reported that 19.2% of individuals with dementia had constipation [27]. Fecal impaction and fecal incontinence are common problems in people with dementia, especially those living in nursing homes [28]. Patients with dementia or Alzheimer’s disease had a 2.6-fold higher risk of laxative use in care homes [29]. The link between dementia and constipation may be attributable to neurocognitive disorders affecting gut–brain interactions, resulting in reduced anorectal sensation and gastrointestinal dysfunction, which lead to delayed colonic transit time [27,30]. Currently no international comparator regarding a biological framework that accounts for population-level variation in gut microbiome composition or colonic transit physiology has been published.

Systemic reviews have summarized that the prevalence of cognitive impairment or dementia among patients undergoing dialysis ranges from 24% to 74% [31,32]. These variable results may be due to differences in study populations, dialysis modalities, and definition of cognitive impairment. Uremic toxins can cause endothelial dysfunction, impaired cerebral autoregulation, increased blood–brain barrier permeability, and adverse effects on neural progenitor cells, thereby diminishes neuronal repair and neurogenesis and ultimately leading to cognitive impairment [33,34]. The prevalence reported in the present study (21.9%) was lower than that reported in previous studies, likely because only patients with dementia, but not those with mild cognitive impairment, were included. Population-specific genetic variability and vascular risk profiles may also partly explain this. For example, apolipoprotein E gene polymorphisms are associated with both Alzheimer’s dementia and end-stage renal disease [52], and the distribution of APOE alleles are different worldwide [53]. Large variations exist in the attributable risk factors for cardiovascular disease, even among countries at similar levels of development [54]. Varied vascular risk profiles may influence the prevalence of dialysis and dementia. A previous meta-analysis concluded that peritoneal dialysis has better cognitive outcomes than hemodialysis [31]. However, due to the relatively small number of cases, hemodialysis and peritoneal dialysis were not analyzed separately in this study.

Higher frequency of all neuropsychiatric symptoms, except for wandering, was observed in patients receiving any form of advanced care. Anxiety, depression, and poor sleep quality have previously been reported among patients with chronic pain, functional constipation, chronic respiratory disease, and dialysis [[35], [36], [37], [38]]. Individuals with psychosis have an increased risk of chronic renal failure and report higher levels of pain [39,40]. Tube feeding, chronic pain, and respiratory care have been associated with agitation and resistance to care [41,42]. It is therefore unsurprising that patients receiving advanced care exhibited more mood disturbances, psychosis, and abnormal behaviors in the present study. Non-pharmacological interventions, such as adequate care partner’s training, pain management, bowel training, daily activity planning, and cognitive stimulation, should be provided to reduce these neuropsychiatric symptoms [11,40,42].

Among people with dementia in the present survey, day–night confusion and care-resistant behavior were significantly more prevalent in the advanced care group. Hsieh et al. also found that the two most common BPSD among institutionalized dementia residents in Taiwan were nighttime behavior (17.9%) and resistance against care (13.4%) [7]. Residents in nursing homes received more advanced care than those still living at home, which may explain the similar findings in both studies [24]. Fauth et al. reported two-thirds of non-institutionalized persons with dementia exhibit care-resistance behavior, which significantly increases care partner’s loading and depression [43]. Disruptions of rest-activity rhythms are frequent in Alzheimer’s disease, particularly in more severe stages, and are among the most common (80%) non-motor symptoms in Parkinson’s disease [44]. More than half of patients with circadian rhythm disturbances have been reported among those with end-stage renal disease and obstructive lung disease [45,46]. Circadian control also modulated pain and neuroinflammation [47]. The present study found that day–night confusion or nighttime behavior was a frequent BPSD (42%) in patients receiving advanced care, particularly in those receiving urinary catheterization or single catheterization. Strategies for resynchronizing the sleep–wake rhythm, such as light therapy or exogenous melatonin, may be incorporated into care plans. For dialysis patients, exogenous erythropoietin, lowering dialysate temperature, and nocturnal hemodialysis represent additional effective interventions [45]. This study also found that care-resistance behavior was prevalent in patients receiving tube feeding (27%). Although only a small number of patients were receiving dialysis, hallucination and aberrant motor behavior may be more prevalent than in those without any advanced care. Previous study also found that 33.7% of patients with chronic kidney disease had neuropsychiatric symptoms, which may be associated with neuroinflammation [56,57]. Person-centered care, music intervention, adequate pain control prior to care, and ability-focused interventions constitute suitable non-pharmacological treatments for individual exhibiting care-resistance behavior [48].

There are several strengths of the present study. First, the study utilized a large-scale, nationally representative epidemiologic dataset on dementia in Taiwan. Second, most of the participants’ dementia diagnoses were confirmed by psychiatrists or neurologists through home-visits. Third, this is the first study to compare prevalence of dementia and BPSD under specific advanced care conditions. Limitations of the study are listed below. First, the cross-sectional design makes the causal relationship between advanced care and dementia difficult to establish. Second, there is a relatively high percentage of participants who did not complete the phase 2 survey due to COVID-19 pandemic. The selection bias may cause an underestimation of dementia prevalence since the most severely ill patients have a higher chance of refusing a physician’s home visit. Dementia severity, such as CDR, is also missing in this population and cannot be incorporate into subgroup analysis. However, dementia diagnoses in these cases were made by an expert consensus panel. Third, the subtypes of dementia are lacking because no neuroimages or fluid biomarkers were used in the survey. The study results are unable to respond to most of pathways mentioned in the discussion section, which are biologically plausible for specific dementia subtypes. Fourth, the study results may be difficult to generalize to other countries due to the specific culture, long-term care and healthcare system in Taiwan. Fifth, the BPSD tool used in the present study relies on care partner’s reports, which may be subject to reporting bias. The tool also lacks cross-cultural validity and may not suitable to generate to other regions. Sixth, the non-significant association between pain care and dementia prevalence may be related to the diversity of pain care. The association between different pain care modalities and the prevalence of dementia needs further study.

In conclusion, the study found that patients receiving advanced care were associated with a significantly higher prevalence of dementia. Ranking from high to low were stoma care or enema care, tube feeding, dialysis, urinary catheterization, and respiratory care. Patients receiving advanced care exhibited higher rates of neuropsychiatric symptoms. Among people with dementia receiving advanced care, day–night confusion and care-resistant behavior were more pronounced. Healthcare professionals should consider the possibility of comorbid dementia in patients receiving advanced care, and provide more patient-centered strategies or additional non-pharmacological interventions alongside pharmacotherapy to alleviate BPSD and reduce care partner’s loading.

## Fundings

The authors are grateful to the Ministry of Health and Welfare, Taiwan, for funding this project (09D1-PHMOHW04, awarded to Dr. Chih-Cheng Hsu).

## Declaration of the use of generative AI and AI-assisted technologies in scientific writing and in figures, images and artwork

The authors declare there were no use of AI in writing and in figures or tables

## CRediT authorship contribution statement

**Kai-Ming Jhang:** Writing – review & editing, Writing – original draft, Validation, Conceptualization. **Yu-Chun Tung:** Writing – review & editing, Writing – original draft, Validation. **Wen-Fu Wang:** Writing – review & editing, Visualization. **Hsiang-Ju Chung:** Writing – review & editing, Investigation, Formal analysis. **Hui-Yi Liao:** Writing – review & editing, Investigation, Formal analysis. **Ling-Chun Huang:** Writing – review & editing, Validation. **Chih-Cheng Hsu:** Writing – review & editing, Conceptualization. **Yuan-Han Yang:** Writing – review & editing, Conceptualization.

## Declaration of competing interest

The authors declare that they have no known competing financial interests or personal relationships that could have appeared to influence the work reported in this paper.

## Data Availability

The raw dataset analyzed in the study is available from the Department of Long-term Care, Ministry of Health and Welfare, Taiwan, on reasonable request.
